# Diphenyl (2-chloro­benzyl­amido)­phosphate

**DOI:** 10.1107/S160053681004955X

**Published:** 2010-12-04

**Authors:** Mehrdad Pourayoubi, Poorya Zargaran, Arnold L. Rheingold, James A. Golen

**Affiliations:** a Department of Chemistry, Ferdowsi University of Mashhad, Mashhad, 91779, Iran; b Department of Chemistry, University of California, San Diego, 9500 Gilman, Drive, La Jolla, CA 92093, USA

## Abstract

In the title compound, C_19_H_17_ClNO_3_P, the P atom exhibits a distorted tetra­hedral configuration. In the crystal, pairs of inter­molecular N—H⋯O(P) hydrogen bonds form centrosymmetric dimers.

## Related literature

For related structures, see: Pourayoubi & Zargaran (2010 ▶); Pourayoubi *et al.* (2010 ▶).
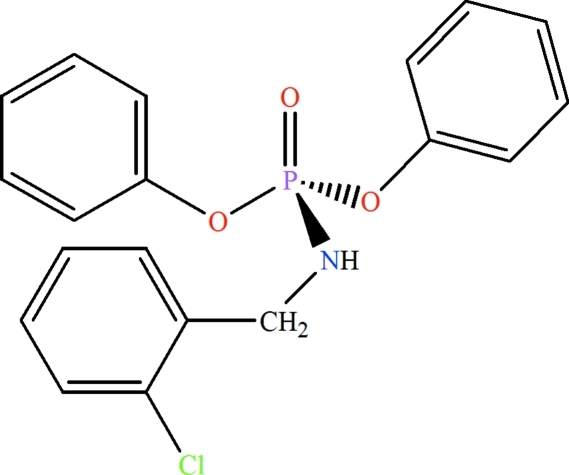


## Experimental

### 

#### Crystal data

C_19_H_17_ClNO_3_P
*M* *_r_* = 373.76
Triclinic, 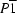

*a* = 8.6178 (5) Å
*b* = 9.5901 (6) Å
*c* = 12.1543 (7) Å
α = 107.609 (1)°
β = 93.882 (1)°
γ = 110.036 (1)°
*V* = 882.86 (9) Å^3^
*Z* = 2
Mo *K*α radiation
μ = 0.33 mm^−1^
*T* = 100 K
0.40 × 0.35 × 0.25 mm

#### Data collection

Bruker APEXII CCD diffractometer
Absorption correction: multi-scan (*SADABS*; Bruker, 2009 ▶) *T*_min_ = 0.881, *T*_max_ = 0.923
13560 measured reflections
3982 independent reflections
3681 reflections with *I* > 2σ(*I*)
*R*_int_ = 0.026

#### Refinement

*R*[*F* ^2^ > 2σ(*F* ^2^)] = 0.033
*wR*(*F* ^2^) = 0.086
*S* = 1.02
3982 reflections
230 parameters
H atoms treated by a mixture of independent and constrained refinement
Δρ_max_ = 0.28 e Å^−3^
Δρ_min_ = −0.44 e Å^−3^

### 

Data collection: *APEX2* (Bruker, 2009 ▶); cell refinement: *SAINT* (Bruker, 2009 ▶); data reduction: *SAINT*; program(s) used to solve structure: *SHELXS97* (Sheldrick, 2008 ▶); program(s) used to refine structure: *SHELXL97* (Sheldrick, 2008 ▶); molecular graphics: *SHELXTL* (Sheldrick, 2008 ▶); software used to prepare material for publication: *SHELXTL*.

## Supplementary Material

Crystal structure: contains datablocks I, global. DOI: 10.1107/S160053681004955X/lh5173sup1.cif

Structure factors: contains datablocks I. DOI: 10.1107/S160053681004955X/lh5173Isup2.hkl

Additional supplementary materials:  crystallographic information; 3D view; checkCIF report

## Figures and Tables

**Table 1:** Hydrogen-bond geometry (Å, °)

*D*—H⋯*A*	*D*—H	H⋯*A*	*D*⋯*A*	*D*—H⋯*A*
N1—H1*N*⋯O1^i^	0.80 (2)	2.08 (2)	2.8703 (15)	172.2 (19)

Symmetry code: (i) 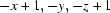.

**Fig. 1.**
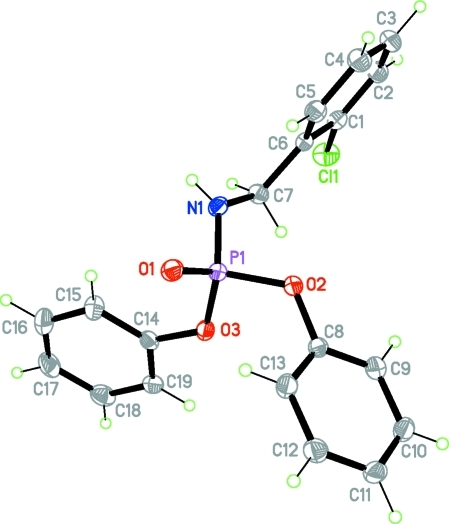
The molecular structure of the title compound. Ellipsoids are given at the 50% probability level.

## supplementary crystallographic information

### Comment

In our previous works, the crystal structures of some amidophosphoric acid ester compounds having the P(O)(OC_6_H_5_)_2_ phosphoester moiety have been reported (Pourayoubi *et al.*, 2010; Pourayoubi & Zargaran, 2010). Herein, we report the synthesis and crystal structure of the title amidophosphoric acid ester.

The molecular structure of the title compound is shown in Fig. 1. The P atom has a distorted tetrahedral configuration with the bond angles in the range of 98.03 (5)° [O2–P1–O3] to 116.37 (6)° [O1–P1–O2]. In the crystal structure, pairs of intermolecular N-H···O(P) hydrogen bonds form centrosymmetric dimers.

### Experimental

To a solution of (C_6_H_5_O)_2_P(O)Cl in chloroform, a solution of 2-chlorobenzylamine (1:2 mole ratio) in chloroform was added at 273 K. After 4 h stirring, the solvent was removed and product was washed with distilled water and recrystallized from CH_3_CN at room temperature. IR (KBr, cm^-1^): 3206.6, 3065.7, 2909.2, 2715.0, 1947.0, 1591.1, 1486.9, 1456.4, 1257.1, 1198.0, 1131.5, 1016.5, 940.3, 756.3, 686.0.

### Refinement

Data corrected for absorption using *SADABS* (Bruker, 2009) and structure solved by direct methods. All non-hydrogen atoms refined as anisotropic by Fourier full matrix least squares. Hydrogen atoms H1N found from a Fourier difference map and allowed to refine while all other hydrogen atoms were placed in calculated positions with appropriate riding models.

### Crystal data

**Table:**

C_19_H_17_ClNO_3_P	*Z* = 2
*M**_r_* = 373.76	*F*(000) = 388
Triclinic, *P*1	*D*_x_ = 1.406 Mg m^−^^3^
Hall symbol: -P 1	Mo *K*α radiation, λ = 0.71073 Å
*a* = 8.6178 (5) Å	Cell parameters from 9988 reflections
*b* = 9.5901 (6) Å	θ = 2.4–28.0°
*c* = 12.1543 (7) Å	µ = 0.33 mm^−^^1^
α = 107.609 (1)°	*T* = 100 K
β = 93.882 (1)°	Block, colourless
γ = 110.036 (1)°	0.40 × 0.35 × 0.25 mm
*V* = 882.86 (9) Å^3^	

### Data collection

**Table:**

Bruker APEXII CCD diffractometer	3982 independent reflections
Radiation source: fine-focus sealed tube	3681 reflections with *I* > 2σ(*I*)
graphite	*R*_int_ = 0.026
φ and ω scans	θ_max_ = 28.0°, θ_min_ = 2.4°
Absorption correction: multi-scan (*SADABS*; Bruker, 2009)	*h* = −11→10
*T*_min_ = 0.881, *T*_max_ = 0.923	*k* = −11→12
13560 measured reflections	*l* = −15→11

### Refinement

**Table:**

Refinement on *F*^2^	Primary atom site location: structure-invariant direct methods
Least-squares matrix: full	Secondary atom site location: difference Fourier map
*R*[*F*^2^ > 2σ(*F*^2^)] = 0.033	Hydrogen site location: inferred from neighbouring sites
*wR*(*F*^2^) = 0.086	H atoms treated by a mixture of independent and constrained refinement
*S* = 1.02	*w* = 1/[σ^2^(*F*_o_^2^) + (0.0396*P*)^2^ + 0.4993*P*] where *P* = (*F*_o_^2^ + 2*F*_c_^2^)/3
3982 reflections	(Δ/σ)_max_ = 0.004
230 parameters	Δρ_max_ = 0.28 e Å^−^^3^
0 restraints	Δρ_min_ = −0.44 e Å^−^^3^

### Special details

**Table:**

Geometry. All e.s.d.'s (except the e.s.d. in the dihedral angle between two l.s. planes) are estimated using the full covariance matrix. The cell e.s.d.'s are taken into account individually in the estimation of e.s.d.'s in distances, angles and torsion angles; correlations between e.s.d.'s in cell parameters are only used when they are defined by crystal symmetry. An approximate (isotropic) treatment of cell e.s.d.'s is used for estimating e.s.d.'s involving l.s. planes.
Refinement. Refinement of *F*^2^ against ALL reflections. The weighted *R*-factor *wR* and goodness of fit *S* are based on *F*^2^, conventional *R*-factors *R* are based on *F*, with *F* set to zero for negative *F*^2^. The threshold expression of *F*^2^ > σ(*F*^2^) is used only for calculating *R*-factors(gt) *etc*. and is not relevant to the choice of reflections for refinement. *R*-factors based on *F*^2^ are statistically about twice as large as those based on *F*, and *R*- factors based on ALL data will be even larger.

### Fractional atomic coordinates and isotropic or equivalent isotropic displacement parameters (Å^2^)

**Table:**

	*x*	*y*	*z*	*U*_iso_*/*U*_eq_	
Cl1	1.12274 (4)	0.65811 (4)	0.77058 (3)	0.02847 (10)	
P1	0.49017 (4)	0.15695 (4)	0.67814 (3)	0.01573 (9)	
O1	0.35884 (12)	0.01133 (11)	0.59500 (8)	0.0201 (2)	
O2	0.44078 (12)	0.30686 (11)	0.72227 (8)	0.01837 (19)	
O3	0.53917 (12)	0.14595 (11)	0.80359 (8)	0.0184 (2)	
N1	0.65627 (15)	0.21809 (13)	0.62570 (10)	0.0194 (2)	
C1	0.97088 (17)	0.61883 (16)	0.65109 (11)	0.0190 (3)	
C2	1.00000 (19)	0.72582 (16)	0.59229 (13)	0.0244 (3)	
H2A	1.1015	0.8170	0.6159	0.029*	
C3	0.8788 (2)	0.69789 (18)	0.49836 (13)	0.0276 (3)	
H3A	0.8965	0.7708	0.4577	0.033*	
C4	0.7317 (2)	0.56346 (18)	0.46398 (13)	0.0262 (3)	
H4A	0.6485	0.5443	0.3999	0.031*	
C5	0.70658 (17)	0.45701 (16)	0.52345 (12)	0.0207 (3)	
H5A	0.6058	0.3650	0.4989	0.025*	
C6	0.82539 (16)	0.48181 (15)	0.61804 (11)	0.0167 (2)	
C7	0.80201 (17)	0.36547 (15)	0.68278 (11)	0.0188 (3)	
H7A	0.9043	0.3410	0.6882	0.023*	
H7B	0.7890	0.4157	0.7639	0.023*	
C8	0.32185 (16)	0.31673 (15)	0.79467 (11)	0.0170 (3)	
C9	0.35893 (18)	0.46128 (16)	0.88220 (12)	0.0206 (3)	
H9A	0.4608	0.5481	0.8920	0.025*	
C10	0.24451 (19)	0.47717 (17)	0.95549 (12)	0.0232 (3)	
H10A	0.2686	0.5755	1.0163	0.028*	
C11	0.09537 (19)	0.35066 (17)	0.94059 (13)	0.0235 (3)	
H11A	0.0181	0.3619	0.9915	0.028*	
C12	0.05959 (18)	0.20772 (17)	0.85097 (13)	0.0241 (3)	
H12A	−0.0430	0.1213	0.8402	0.029*	
C13	0.17259 (18)	0.18976 (16)	0.77676 (12)	0.0215 (3)	
H13A	0.1478	0.0921	0.7149	0.026*	
C14	0.60296 (16)	0.03684 (15)	0.82152 (12)	0.0177 (3)	
C15	0.60994 (18)	−0.08832 (16)	0.73025 (13)	0.0233 (3)	
H15A	0.5699	−0.1047	0.6508	0.028*	
C16	0.67701 (19)	−0.18925 (17)	0.75786 (14)	0.0277 (3)	
H16A	0.6826	−0.2756	0.6965	0.033*	
C17	0.7356 (2)	−0.16551 (18)	0.87353 (15)	0.0287 (3)	
H17A	0.7817	−0.2348	0.8913	0.034*	
C18	0.7269 (2)	−0.03992 (19)	0.96360 (14)	0.0278 (3)	
H18A	0.7667	−0.0235	1.0431	0.033*	
C19	0.65988 (18)	0.06176 (16)	0.93767 (12)	0.0214 (3)	
H19A	0.6533	0.1475	0.9991	0.026*	
H1N	0.661 (2)	0.160 (2)	0.5644 (18)	0.031 (5)*	

### Atomic displacement parameters (Å^2^)

**Table:**

	*U*^11^	*U*^22^	*U*^33^	*U*^12^	*U*^13^	*U*^23^
Cl1	0.02121 (18)	0.0312 (2)	0.02217 (17)	0.00205 (14)	−0.00251 (13)	0.00503 (14)
P1	0.01745 (17)	0.01436 (16)	0.01548 (16)	0.00594 (13)	0.00454 (12)	0.00518 (12)
O1	0.0195 (5)	0.0182 (5)	0.0192 (4)	0.0047 (4)	0.0049 (4)	0.0043 (4)
O2	0.0209 (5)	0.0169 (4)	0.0210 (4)	0.0091 (4)	0.0081 (4)	0.0086 (4)
O3	0.0242 (5)	0.0170 (4)	0.0164 (4)	0.0107 (4)	0.0044 (4)	0.0056 (3)
N1	0.0207 (6)	0.0157 (5)	0.0184 (5)	0.0048 (4)	0.0074 (5)	0.0029 (4)
C1	0.0186 (6)	0.0199 (6)	0.0170 (6)	0.0080 (5)	0.0041 (5)	0.0036 (5)
C2	0.0262 (7)	0.0184 (6)	0.0290 (7)	0.0072 (6)	0.0116 (6)	0.0088 (5)
C3	0.0369 (8)	0.0268 (7)	0.0302 (7)	0.0179 (7)	0.0142 (7)	0.0170 (6)
C4	0.0302 (8)	0.0322 (8)	0.0235 (7)	0.0178 (6)	0.0053 (6)	0.0128 (6)
C5	0.0198 (6)	0.0218 (6)	0.0201 (6)	0.0088 (5)	0.0032 (5)	0.0059 (5)
C6	0.0181 (6)	0.0166 (6)	0.0166 (6)	0.0086 (5)	0.0054 (5)	0.0049 (5)
C7	0.0178 (6)	0.0189 (6)	0.0188 (6)	0.0054 (5)	0.0025 (5)	0.0072 (5)
C8	0.0190 (6)	0.0198 (6)	0.0173 (6)	0.0109 (5)	0.0048 (5)	0.0091 (5)
C9	0.0211 (7)	0.0179 (6)	0.0223 (6)	0.0072 (5)	0.0035 (5)	0.0068 (5)
C10	0.0289 (7)	0.0217 (7)	0.0209 (6)	0.0132 (6)	0.0057 (6)	0.0056 (5)
C11	0.0259 (7)	0.0295 (7)	0.0249 (7)	0.0171 (6)	0.0107 (6)	0.0140 (6)
C12	0.0199 (7)	0.0238 (7)	0.0315 (7)	0.0085 (5)	0.0084 (6)	0.0125 (6)
C13	0.0217 (7)	0.0185 (6)	0.0238 (7)	0.0085 (5)	0.0050 (5)	0.0056 (5)
C14	0.0161 (6)	0.0162 (6)	0.0225 (6)	0.0057 (5)	0.0050 (5)	0.0092 (5)
C15	0.0257 (7)	0.0201 (6)	0.0231 (7)	0.0103 (6)	0.0016 (5)	0.0049 (5)
C16	0.0281 (8)	0.0198 (7)	0.0352 (8)	0.0120 (6)	0.0052 (6)	0.0066 (6)
C17	0.0272 (8)	0.0265 (7)	0.0410 (9)	0.0138 (6)	0.0075 (7)	0.0193 (7)
C18	0.0297 (8)	0.0339 (8)	0.0279 (7)	0.0142 (6)	0.0068 (6)	0.0190 (6)
C19	0.0230 (7)	0.0229 (7)	0.0214 (6)	0.0094 (5)	0.0080 (5)	0.0102 (5)

### Geometric parameters (Å, °)

**Table:**

Cl1—C1	1.7429 (14)	C8—C9	1.3834 (18)
P1—O1	1.4699 (10)	C8—C13	1.3831 (19)
P1—O2	1.5872 (9)	C9—C10	1.389 (2)
P1—O3	1.5984 (9)	C9—H9A	0.9500
P1—N1	1.6042 (12)	C10—C11	1.387 (2)
O2—C8	1.4043 (15)	C10—H10A	0.9500
O3—C14	1.4002 (15)	C11—C12	1.386 (2)
N1—C7	1.4591 (17)	C11—H11A	0.9500
N1—H1N	0.80 (2)	C12—C13	1.390 (2)
C1—C2	1.3850 (19)	C12—H12A	0.9500
C1—C6	1.3942 (18)	C13—H13A	0.9500
C2—C3	1.389 (2)	C14—C19	1.3829 (19)
C2—H2A	0.9500	C14—C15	1.3884 (19)
C3—C4	1.388 (2)	C15—C16	1.392 (2)
C3—H3A	0.9500	C15—H15A	0.9500
C4—C5	1.388 (2)	C16—C17	1.384 (2)
C4—H4A	0.9500	C16—H16A	0.9500
C5—C6	1.3910 (18)	C17—C18	1.389 (2)
C5—H5A	0.9500	C17—H17A	0.9500
C6—C7	1.5175 (17)	C18—C19	1.389 (2)
C7—H7A	0.9900	C18—H18A	0.9500
C7—H7B	0.9900	C19—H19A	0.9500
			
O1—P1—O2	116.37 (6)	C9—C8—O2	116.66 (12)
O1—P1—O3	113.92 (5)	C13—C8—O2	121.59 (12)
O2—P1—O3	98.03 (5)	C8—C9—C10	118.77 (13)
O1—P1—N1	113.05 (6)	C8—C9—H9A	120.6
O2—P1—N1	104.00 (6)	C10—C9—H9A	120.6
O3—P1—N1	110.13 (6)	C11—C10—C9	120.55 (13)
C8—O2—P1	123.42 (8)	C11—C10—H10A	119.7
C14—O3—P1	124.66 (8)	C9—C10—H10A	119.7
C7—N1—P1	125.99 (9)	C12—C11—C10	119.65 (13)
C7—N1—H1N	117.4 (14)	C12—C11—H11A	120.2
P1—N1—H1N	116.5 (14)	C10—C11—H11A	120.2
C2—C1—C6	122.43 (13)	C11—C12—C13	120.56 (14)
C2—C1—Cl1	118.35 (11)	C11—C12—H12A	119.7
C6—C1—Cl1	119.22 (10)	C13—C12—H12A	119.7
C1—C2—C3	119.07 (13)	C8—C13—C12	118.73 (13)
C1—C2—H2A	120.5	C8—C13—H13A	120.6
C3—C2—H2A	120.5	C12—C13—H13A	120.6
C4—C3—C2	119.95 (13)	C19—C14—C15	121.49 (12)
C4—C3—H3A	120.0	C19—C14—O3	115.36 (11)
C2—C3—H3A	120.0	C15—C14—O3	123.15 (12)
C3—C4—C5	119.83 (14)	C14—C15—C16	118.46 (13)
C3—C4—H4A	120.1	C14—C15—H15A	120.8
C5—C4—H4A	120.1	C16—C15—H15A	120.8
C4—C5—C6	121.60 (13)	C17—C16—C15	120.86 (14)
C4—C5—H5A	119.2	C17—C16—H16A	119.6
C6—C5—H5A	119.2	C15—C16—H16A	119.6
C5—C6—C1	117.10 (12)	C16—C17—C18	119.76 (13)
C5—C6—C7	122.47 (12)	C16—C17—H17A	120.1
C1—C6—C7	120.42 (12)	C18—C17—H17A	120.1
N1—C7—C6	113.03 (11)	C19—C18—C17	120.15 (14)
N1—C7—H7A	109.0	C19—C18—H18A	119.9
C6—C7—H7A	109.0	C17—C18—H18A	119.9
N1—C7—H7B	109.0	C14—C19—C18	119.28 (13)
C6—C7—H7B	109.0	C14—C19—H19A	120.4
H7A—C7—H7B	107.8	C18—C19—H19A	120.4
C9—C8—C13	121.71 (13)		
			
O1—P1—O2—C8	67.62 (11)	C1—C6—C7—N1	−171.04 (11)
O3—P1—O2—C8	−54.18 (10)	P1—O2—C8—C9	140.36 (10)
N1—P1—O2—C8	−167.34 (10)	P1—O2—C8—C13	−41.74 (16)
O1—P1—O3—C14	59.85 (11)	C13—C8—C9—C10	1.6 (2)
O2—P1—O3—C14	−176.56 (10)	O2—C8—C9—C10	179.53 (11)
N1—P1—O3—C14	−68.38 (11)	C8—C9—C10—C11	−0.4 (2)
O1—P1—N1—C7	174.57 (10)	C9—C10—C11—C12	−0.7 (2)
O2—P1—N1—C7	47.45 (12)	C10—C11—C12—C13	0.6 (2)
O3—P1—N1—C7	−56.72 (12)	C9—C8—C13—C12	−1.7 (2)
C6—C1—C2—C3	1.3 (2)	O2—C8—C13—C12	−179.47 (12)
Cl1—C1—C2—C3	−178.73 (11)	C11—C12—C13—C8	0.5 (2)
C1—C2—C3—C4	−0.6 (2)	P1—O3—C14—C19	171.04 (10)
C2—C3—C4—C5	−0.2 (2)	P1—O3—C14—C15	−8.87 (18)
C3—C4—C5—C6	0.4 (2)	C19—C14—C15—C16	−0.4 (2)
C4—C5—C6—C1	0.29 (19)	O3—C14—C15—C16	179.53 (13)
C4—C5—C6—C7	−178.79 (12)	C14—C15—C16—C17	−0.1 (2)
C2—C1—C6—C5	−1.16 (19)	C15—C16—C17—C18	0.4 (2)
Cl1—C1—C6—C5	178.91 (10)	C16—C17—C18—C19	−0.2 (2)
C2—C1—C6—C7	177.94 (12)	C15—C14—C19—C18	0.5 (2)
Cl1—C1—C6—C7	−1.99 (17)	O3—C14—C19—C18	−179.36 (12)
P1—N1—C7—C6	−113.96 (12)	C17—C18—C19—C14	−0.3 (2)
C5—C6—C7—N1	8.01 (18)		

### Hydrogen-bond geometry (Å, °)

**Table:**

*D*—H···*A*	*D*—H	H···*A*	*D*···*A*	*D*—H···*A*
N1—H1N···O1^i^	0.80 (2)	2.08 (2)	2.8703 (15)	172.2 (19)

Symmetry codes: (i) −*x*+1, −*y*, −*z*+1.
